# Evolutional Characterization of Photochemically Induced Stroke in Rats: a Multimodality Imaging and Molecular Biological Study

**DOI:** 10.1007/s12975-016-0512-4

**Published:** 2016-12-01

**Authors:** Nai-Wei Liu, Chien-Chih Ke, Yonghua Zhao, Yi-An Chen, Kim-Chuan Chan, David Tat-Wei Tan, Jhih-Shian Lee, You-Yin Chen, Tun-Wei Hsu, Ya-Ju Hsieh, Chi-Wei Chang, Bang-Hung Yang, Wen-Sheng Huang, Ren-Shyan Liu

**Affiliations:** 1 State Key Laboratory of Quality Research in Chinese Medicine, Faculty of Chinese Medicine, Macau University of Science and Technology, Avenida Wai Long, Taipa 999078 Macau; 2 Biomedical Imaging Research Center, National Yang-Ming University, Taipei, Taiwan; 3 Department of Biomedical Imaging and Radiological Sciences, National Yang-Ming University, Taipei, Taiwan; 4 Institute of Clinical Medicine, National Yang-Ming University, Taipei, Taiwan; 5 Department of Medical Engineering, National Yang-Ming University, Taipei, Taiwan; 6 Department of Radiology, Taipei Veterans General Hospital, Taipei, Taiwan; 7 Department of Biomedical Imaging and Radiological Sciences, Kaohsiung Medical University, Kaohsiung, Taiwan; 8 Molecular and Genetic Imaging Core/Taiwan Mouse Clinic, National Comprehensive Mouse Phenotyping and Drug Testing Center, Taipei, Taiwan; 9 Department of Nuclear Medicine and National PET/Cyclotron Center, Taipei Veterans General Hospital, Taipei, Taiwan

**Keywords:** Photothrombotic stroke, Multimodality imaging

## Abstract

Photochemically induced cerebral ischemia is an easy-manipulated, reproducible, relatively noninvasive, and lesion controllable model for translational study of ischemic stroke. In order to longitudinally investigate the characterization of the model, magnetic resonance imaging, ^18^F-2-deoxy-glucose positron emission tomography, fluorescence, and bioluminescence imaging system were performed in correlation with triphenyl tetrazolium chloride (TTC), hematoxylin-eosin staining, and immunohistochemistry examinations of glial fibrillary acidic protein, CD68, NeuN, von willebrand factor, and α-smooth muscle actin in the infarct zone. The results suggested that the number of inflammatory cells, astrocytes, and neovascularization significantly elevated in peri-infarct region from day 7 and a belt of macrophage/microglial and astrocytes was formed surrounding infarct lesion at day 14. Both vasogenic and cytotoxic edema, as well as blood brain-barrier leakage, occurred since day 1 after stroke induction and gradually attenuated with time. Numerous cells other than neuronal cells infiltrated into infarct lesion, which resulted in no visible TTC negative regional existence at day 14. Furthermore, recovery of cerebral blood flow and glucose utilization in peri-infarct zone were noted and more remarkably than that in infarct core following the stroke progression. In conclusion, these characterizations may be highly beneficial to the development of therapeutic strategies for ischemic stroke.

## Introduction

Cerebral ischemia accounts for 80 % of all human strokes and has a major impact on the public health [1]. It causes primary neuronal death in the ischemic region and leads to delayed neuronal degeneration in the penumbra [2]. Stroke disables the patients more than it kills. This fact has led a recent effort to develop strategies for neural repair after stroke. Several small animal models of stroke have been developed to identify mechanisms of cerebral ischemia for developing novel recanalyzing, neuroprotective, neuroregenerative, or anti-inflammatory drugs at a preclinical level [3, 4]. Among them, middle cerebral artery occlusion (MCAo) by an intraluminal filament technique is the most widely used method [5]. Another approach that is technically simpler involves induction of cerebral ischemia and infarction in the cortical vasculature of rats by photochemical reaction triggered by systemic administration of rose bengal (disodium tetraiodo-tetrachloro-fluorescein) and focal illumination of the brain [6]. Illumination leads to production of singlet oxygen via dye triplet energy transfer, which in turn induces peroxidative damage to the endothelium and a vasoconstriction. Consequently, platelet aggregation is produced, with the development of a thrombus and vascular occlusion, and a distal territory ischemia is formed [6–8]. Rats with small infarct volume induced by this method have low mortality, and even performed on aged rats, the mortality of rats with photothrombotic stroke remained much lower than those with global, permanent stroke [9, 10]. An animal model of cerebral ischemia, providing both reproducibility and precise control of lesion size, is critical for translational studies. MCAo produces large ischemic lesions of varying size, which may be considered as a burden to research conducting. Further, MACo lacks the participation of platelet aggregation which is primary initiator of clinical ischemic events [11]. The photothrombotic model not only overcomes the drawbacks of MCAo but can also be used to characterize inflammatory response and apoptosis following thrombosis [12–14] and to monitor structural and functional plasticity of neurons [15].

Current managements of acute stroke include restoring cerebral blood flow (CBF) to ischemic penumbral area by thrombolytic therapy, interventional procedures, surgery, and/or cell-based therapy to enhance tissue repair and functional recovery after ischemic stroke [16–18]. Noninvasive imaging modalities, such as magnetic resonance imaging (MRI) and positron emission tomography (PET), are promising for investigating the evolution of stroke, confirming the validity of models, monitoring the effect of revascularization interventions, and evaluating the efficacy of novel therapeutic drugs [19–23]. MRI has been used in several preclinical studies of stroke using MCAo model [8, 20, 21]. When ischemic brain injury occurs, glucose metabolism changes in the infarct and peri-infarct regions. This was demonstrated in earlier reports using ^14^C-2-deoxy-D-glucose (^14^C-2-DG) autoradiography for the study of photochemically induced ischemic stroke [24] and MCAo model [25]. However, method of ^14^C-2-DG with autoradiography is limited for in vivo longitudinal evaluation of glucose metabolism on the same animal. Until recently, ^18^F-2-deoxy-glucose (FDG) PET imaging was used to evaluate glucose metabolism in transient and permanent MCAo models of rats [26, 27]. The powerful imaging tools of ^18^F-FDG/PET and MRI offer the information of metabolic changes, lesion structure, edematous and CBF status, respectively. In order to characterize the temporal evolution of stroke lesion induced by photothrombotic method in 14 days, ^18^F-FDG/PET, T2-weighted image (T_2_WI), perfusion-weighted image (PWI), and diffusion-weighted image (DWI) were performed to measure cerebral glucose metabolism, edematous lesion, tissue perfusion, and water diffusion, respectively. This image information was further correlated with the cellular and molecular analysis including tissue viability, morphological changes, inflammatory response, astrocyte scar formation, neovascularization, and blood-brain barrier (BBB) permeability, performed by 2, 3, 5-triphenyl tetrazolium chloride (TTC), hematoxylin-eosin (H&E) staining, immunohistochemistry (IHC) staining, and ex vivo Evans blue (EB) imaging in the photochemically induced stroke model of rat.

## Materials and Methods

### Animals and Study Design

Seven-week-old male Sprague-Dawley rats were kept under standardized condition (12–12-h light-dark cycle, with free access to food and water). The animals received serial MRI and ^18^F-FDG PET/computed tomography (CT) imaging at day 1, 3, 7, and 14 after photothrombotic stroke induction. For TTC, H&E, IHC, and EB staining, rats were sacrificed at day 1, 3, 7, and 14 after stroke induction, and intact brain was carefully removed for subsequent manipulation. All animal experiments in this study were conducted according to the guidelines set by the National Laboratory Animal Center and approved by the Institutional Animal Care and Use Committee of National Yang-Ming University and Macau University of Science and Technology. Reporting of this work complies with Animal Research: Reporting in Vivo Experiments (ARRIVE) guidelines.

### Photochemically Induced Stroke

Rats were temporarily anesthetized with isoflurane (induction 3.0 % in air) followed by intramuscular injection of Dexdomitor and Zoletil 50 mixture (1:1) (Orion, Finland and Virbac, France, respectively) with a dose of 100 μl per 300 g body weight and placed in a stereotaxic frame (Narishige Instruments, Tokyo, Japan). After a small incision was made on the scalp, a craniotomic window (3 mm × 6 mm) was made over the somatosensory cortex with the center at the coordinate of 1 mm rostrally from the bregma and 3.5 mm lateral to the midline. A laser beam of 1.5 mm diameter and 532 nm wavelength (GPD105-M-12, Onset Electro-Optics, Taiwan) was stereotactically positioned at the middle of the craniotomic window and illuminated for 20 min. During the first 2 min of illumination, rose bengal (2 ml/kg body weight, concentration: 10 mg/ml saline) was slowly injected through the tail vein. Two control groups were performed following full procedure except for laser illumination or rose bengal injection. All rats after stroke induction were able to survive until they were sacrificed at the end point in this study.

### Regional Cerebral Vasculature Examination with Laser Speckle Contrast Imaging

Laser speckle contrast imaging (LSCI) is a technique in which coherent light incidence on a surface produces a reflected speckle pattern that is related to the underlying movement of optical scatters, such as red blood cells, indicating blood flow [28]. Before and after thrombosis was induced by photochemical method, regional cerebral vasculature was examined by LSCI. Briefly, a moorFLPI-2 Full-Field Laser Perfusion Imager (Moor Instruments, Axminster, UK) was placed at the center of cranial window on the somatosensory cortex where the laser beam illuminated. Before and after laser illumination, the laser speckle imaging was acquired with 25-Hz sampling frequency, 1 frame/s, 580 × 752 pixels resolution, and zoom size of 5.6 mm × 7.5 mm.

### TTC Assay and H&E Staining

At day 1, 3, 7, and 14 after initiation of photothrombotic stroke, selected rats (*n* = 3 at each day) were sacrificed by overdose injection of pentobarbital for tissue viability and histopathological examinations. Following sacrifice, fresh brain was removed from the skull, washed in iced phosphate buffer saline (PBS), and placed in a brain mold. Coronal sections of 1 mm in thickness were cut through the cerebrum and placed in 2 % TTC (Sigma) for 15 min in a 37 °C incubator for the macroscopic determination of tissue viability. All sections were photographed for delineation of infarct as revealed with TTC. The brain sections were then fixed with 10 % formalin and processed with H&E staining for microscopic examination.

### Immunohistochemical Staining

Paraffin-embedded rat brain tissues with thrombotic stroke at day 1, 3, 7, and 14 (*n* = 3 at each time point) were cut into 5-μm section slices. After 1-h blocking (10 % normal serum, 1 % bovine serum albumin [BSA], and 0.025 % TritonX-100 in tris-HCL buffered solution [TBS]), sections were incubated overnight at 4 °C with the following primary antibodies (pre-diluted in TBS containing 1 % BSA): anti-alpha smooth muscle actin (αSMA, 1:100, ab7817, Abcam), anti-von Willebrand Factor (vWF, 1:100, ab6994, Abcam), anti-glial fibrillary acidic protein (GFAP, 1:200, ab53554, Abcam), anti-CD68 (1:100, mab6564, Abnova), and anti-NeuN (1:200 #52283, Arigobio). After being rinsed with TBS-0.1 % Tween-20, tissue sections were detected using horseradish peroxidase-conjugated secondary antibodies and the DAKO Dual Link system (DAKO, K4065) with 2 % 3,3-diaminobenzidine. The images were photographed by the Aperio Image Scope 12.3 (Leica).

### T_2_WI, DWI, and PWI Examinations by Magnetic Resonance Imaging

BioSpec-70/30 7T system (Bruker, Ettlingen, Germany) using a birdcage head-coil of 75 mm inner diameter for radio frequency (RF) transmission and a 20 mm diameter surface coil for reception was used for MRI experiment. The same rat at day 1, 3, 7, and 14 after thrombotic stroke was anesthetized with initial inhalation of 4 % isoflurane for 3 min and maintained with 2 % isoflurane in a mixture of 20 % oxygen and 80 % room air. Prior to imaging, rats under anesthesia were placed in the stereotaxic holder of MRI machine equipped with a heating system to maintain body temperature and a pressure detector to monitor respiration. MRI data sets consisting TWI, DWI, and PWI were acquired at corresponding time points. T_2_WIs were acquired with a multislice multiecho Carr, Purcell, Meiboom, Gill (CPMG) sequence: repetition time (TR) = 2500 msec, echo time (TE) = 33 msec, 16 echoes, field of view (FOV) = 25 × 25 mm^2^, slice thickness = 1 mm, matrix = 256 × 256. Eight coronal and eight horizontal slices were acquired covering a volume extending 10 mm in the rostrocaudal direction. Coronal slices were centered around the infarction lesion, whereas horizontal slices were aligned with the skullcap. DWIs were recorded with a multislice Stejskal-Tanner spin-echo sequence: TR = 4000 msec, echo time = 22 msec, field of view = 25 × 25 mm^2^, slice thickness = 1 mm, matrix = 128 × 128. Data were recorded in the same coronal and horizontal slices as chosen for T_2_WI. Two sets of images were acquired with two different diffusion-encoding gradient strengths (b = 30, 1000 s/mm^2^) in the rostrocaudal direction. PWIs were performed with pulsed arterial spin labeling (PASL) technique using a flow-sensitive alternating inversion-recovery echo planar imaging (FAIR-EPI) sequence with matrix = 96 × 96, FOV = 25 × 25 mm^2^, inversion recovery time (TIR) = 30 to 2300 msec, number of TIR values = 22, recovery time = 10,000 msec, TE/TR > = 10/18,000 msec. The T_1_ changes between slice selective inversion sequence and nonselective inversion sequence were used for CBF quantification [29]. The infarct volume and CBF ratio were measured by using PMOD software (version 3.0; PMOD Technologies). The CBF difference indexes (CBFDI) of the infarct lesion, peri-infarct region, ipsilateral remote cortex, and hippocampus were calculated as follows: CBFDI = (CBF of the mirror site of contralateral hemisphere–CBF of the selected region in the affected hemisphere)/CBF of the mirror site of contralateral hemisphere.

### Evaluation of Cerebral Glucose Metabolism by MicroPET/CT Imaging

^18^F-FDG-PET/CT was performed to assess the evolutional change of cerebral glucose utilization. After a 12-h fasting, thrombotic stroke rats (*n* = 5) at 1, 3, 7, and 14 days were anesthetized with isoflurane and intravenously administered approximately 37 MBq (~1 mCi) of ^18^F-FDG. Sixty minutes later, microPET/CT images were acquired for 30 min using a FLEX X-PET and X-O small animal imaging system (GE Healthcare) with the spatial resolution of 1.6 mm and the voxel size of 0.4 mm × 0.4 mm × 1.2 mm. CT images were acquired with 256 projections over 2 min for attenuation correction and anatomy landmarks. PET data were reconstructed using 3D ordered subset expectation maximization (OSEM) method. CT images were reconstructed using a cone-beam reconstruction algorithm. PET and CT images were co-registered using commercial software (Visage Imaging) with 72-μm isotropic CT spatial resolution and 2 mm for PET imaging. For quantitative analysis, volume of interests (VOIs) were drawn on the infarct lesion, one-voxel width surrounding the lesion, and remote region on the ipsilateral cortex. VOIs were also selected at the mirror sites of the contralateral hemispheres. Percent-injected dose of ^18^F-FDG per c.c. of brain tissue (%ID/cm^3^) was obtained from each VOI. The metabolic difference index (MDI) of each VOI was calculated with the following formula: MDI = (%ID/c.c. of contralateral side VOI − %ID/c.c. of lesion VOI)/%ID/c.c. of contralateral side VOI.

### Assessment of Blood-Brain Barrier by EB Dye Staining

Disruption of BBB was evaluated at day 1, 3, 7, and 14 after photothrombotic stroke induction. Briefly, 2 h after EB dye (Sigma, 10 mg/ml in saline, 2.5 ml/kg rat weight) was injected via tail vein, animals were sacrificed with an overdose of pentobarbital injection (RMB, Animal Health Ltd., UK). The brains were then removed from the skulls immediately after death. To detect the presence of EB, the intact brain was imaged using in vivo fluorescence and bioluminescence imaging system (IVIS 50, Perkin Elma, UK) with following steps and image acquisition settings: the brains were placed at the center of imaging field, and images were acquired for 2 s using Cy5.5 band pass filter channel (excitation/emission wavelength: 615~665 nm/695~770 nm). Region of interest (ROI) selection and quantification were performed using living image software 3.2 (IVIS Imaging System, Perkin Elma, UK).

### Statistical Analysis

The numerical data were reported as means ± standard deviation (SD). Statistical analysis was carried out with the SPSS for windows software package (release 13.0, SPSS Inc., Chicago IL). One-way ANOVA and Tukey post hoc test were used to compare the MDIs and fluorescent signal intensity of EB staining between different time points after stroke. A significant difference was considered if the *p* value was less than 0.05.

## Results

### Cerebral Vascular Occlusion by Photothrombotic Induction

**Fig. 1:**
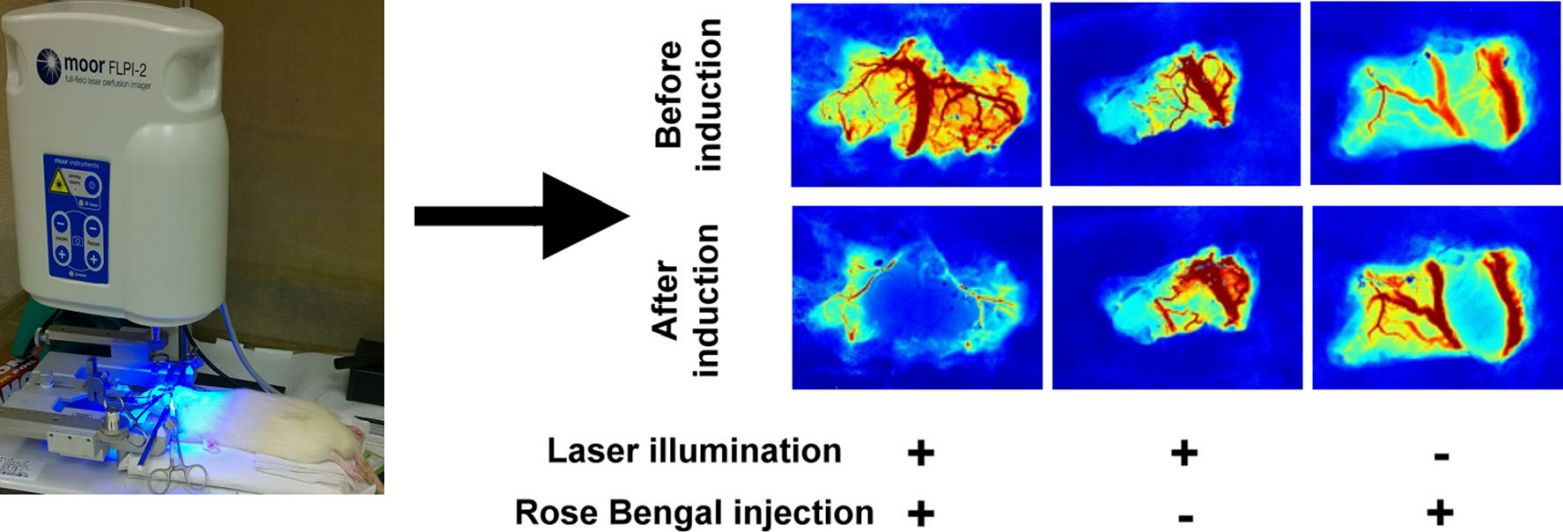
Laser speckle contrast imaging (LSCI) before and after photochemical induction of stroke. Cerebral vasculature occlusion was presented after photothrombotic stroke. Either illumination of laser beam or sole injection of rose bengal did not block cerebral blood flow as shown on LSCI

Photochemical induction of embolic stroke was completed by 532-nm LASER illumination at somatosensory cortex through the cranial window upon rose bengal infusion. Vasoconstriction and blockade of blood flow were examined by LSCI before and after stroke induction. As shown in Fig. 1, the surface vessel network was clearly appeared before induction in each group. Thirty minutes after stroke induction, remarkably vanishing of blood vessel network was observed in rats injected with rose bengal through the cranial window where the LASER beam illuminated at, while hyperemic blood vessel network was presented in the group of only illuminated by laser beam and there was no change of blood flow in the group of sole injection of rose bengal. The rapid and massive coagulation of vessels within the illumination area features the pathomechanism of this method which is not similar to that in clinical ischemic stroke usually caused by an embolus or two.

### Tissue Viability and Morphological Characteristics of Infarct Lesion

**Fig. 2:**
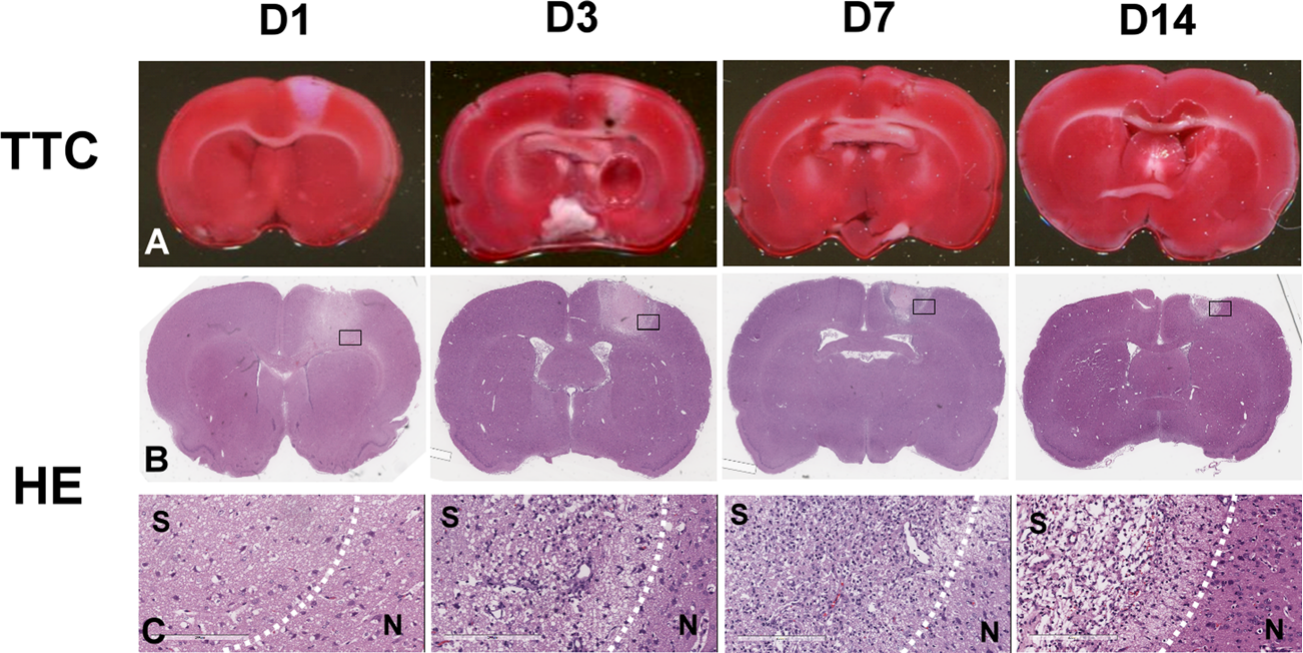
Longitudinal TTC and H&E staining at day 1, 3, 7, and 14 after induction of photochemical thrombosis. TTC-negative area, observed at the site where laser illuminated, was largest in size at day 1, then decreased with time, and was almost not detectable at day 14 (**a**). **b**–**c** Morphological change in H&E-stained tissue sections. It showed a typical stroke-induced liquefactive necrosis at day 1, dilated vessels at day 3, infiltration of cells into the surrounding area of ischemia at days 3 and 7, and neovascular formation at day 14

Tissue viability and morphologic change of the photochemically induced stroke were evaluated by TTC and H&E stains at day 1, 3, 7, and 14 post-induction. Results of TTC and H&E staining at each time point were shown in Fig. 2. TTC-negative region which represents the necrotic and non-viable tissue was well demonstrated on the first day after stroke induction, and gradually decreased in size afterward. At day 3, in addition to the shrinkage of ischemic lesion, the boundary between TTC-positive and TTC-negative regions became blurred, as compared to a sharp, clear dividing line at day 1, indicating an increasing number of cells which were migrating and infiltrating to the ischemic margin from outside the region. The volume of the infarct lesions at day 3 to day 14 reduced gradually and remarkably compared to that of the initial infarct volume at day 1. TTC-negative region became hardly observed at day 14 (Fig. 2a). Microscopic examination of the H&E-stained tissues showed a typical stroke-induced liquefactive necrosis at day 1, dilated vessels at day 3, infiltration of cells into the surrounding area of ischemia at days 3 and 7, and neovascular formation at day 14 (Fig. 2b, c).

### The Change of Inflammatory Cells and Astrocytes in Infarct Region

**Fig. 3:**
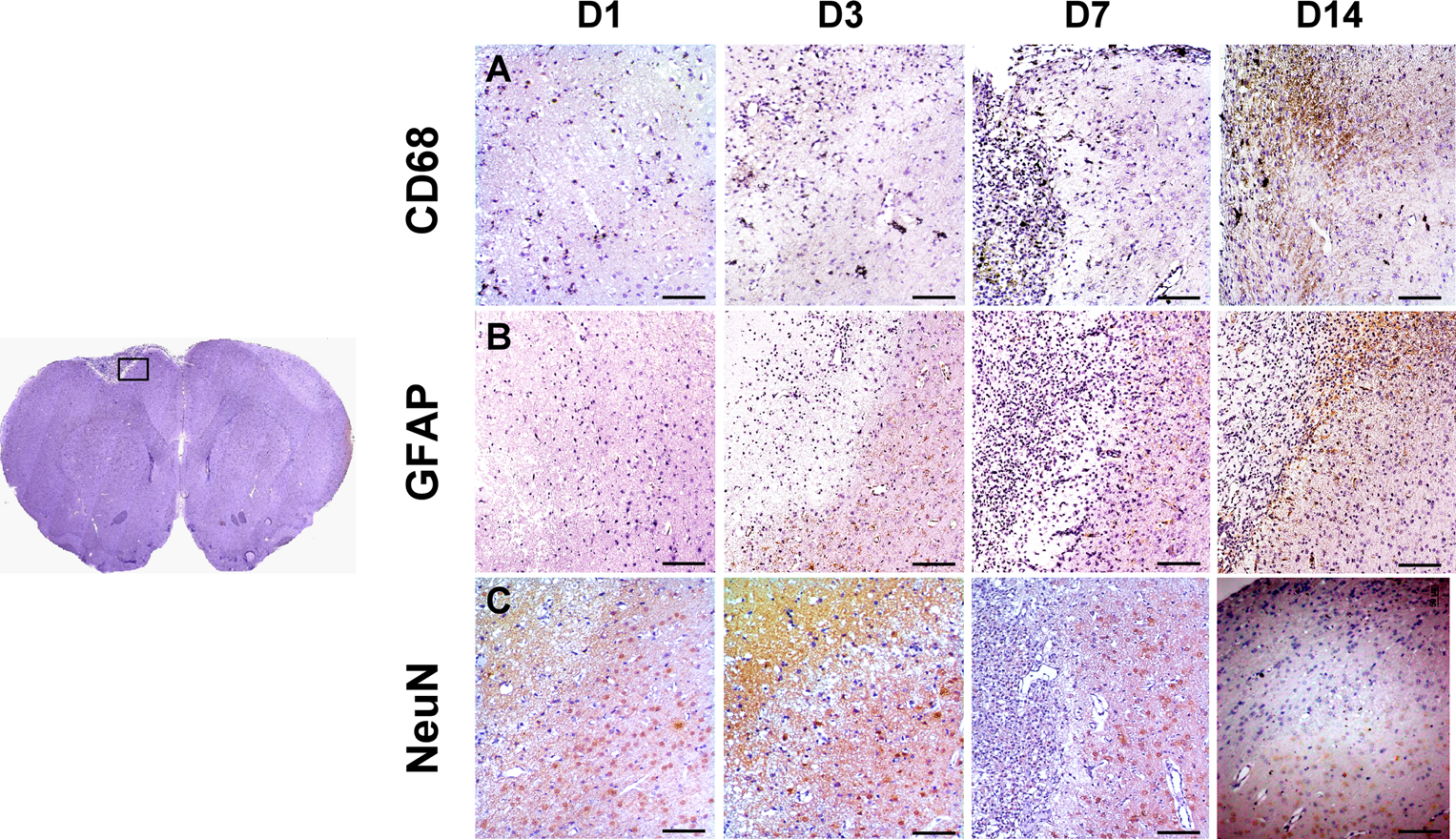
Immunohistochemical staining of GFAP, CD68, and NeuN at day 1, 3, 7, and 14 after stroke induction. At day 7 post-stroke, the result of staining indicated that GFAP- and CD68-positive cells accumulated at the peri-infarct zone and formed a belt surrounding the infarct lesion at day 14. No NeuN expression was observed in ischemic zone throughout 14 days. *Scale bar* 100 μm

As a marker of macrophage and microglia, CD68 expression represents the evolution of inflammatory response post-stroke [30]. Figure 3a shows the presence of CD68-positive cells in the border zone of infarction lesion at day 7, and the cell number increased in the lesion at day 14. IHC staining using antibody against GFAP showed the infiltration of astrocytes into infarct boundary at day 7, and interdigitating compact astrocyte scar surrounding the lesion core was observed at day 14 (Fig. 3b). In addition, NeuN-expressing mature neuronal cells were absent in stroke region throughout 14 days after induction (Fig. 3c). These results indicated that the cellular evolutional changes in the lesion were mainly the inflammatory response and astrocyte scar formation, instead of neurogenesis.

### Angiogenesis and Vasculogenesis in Infarct Lesion

**Fig. 4:**
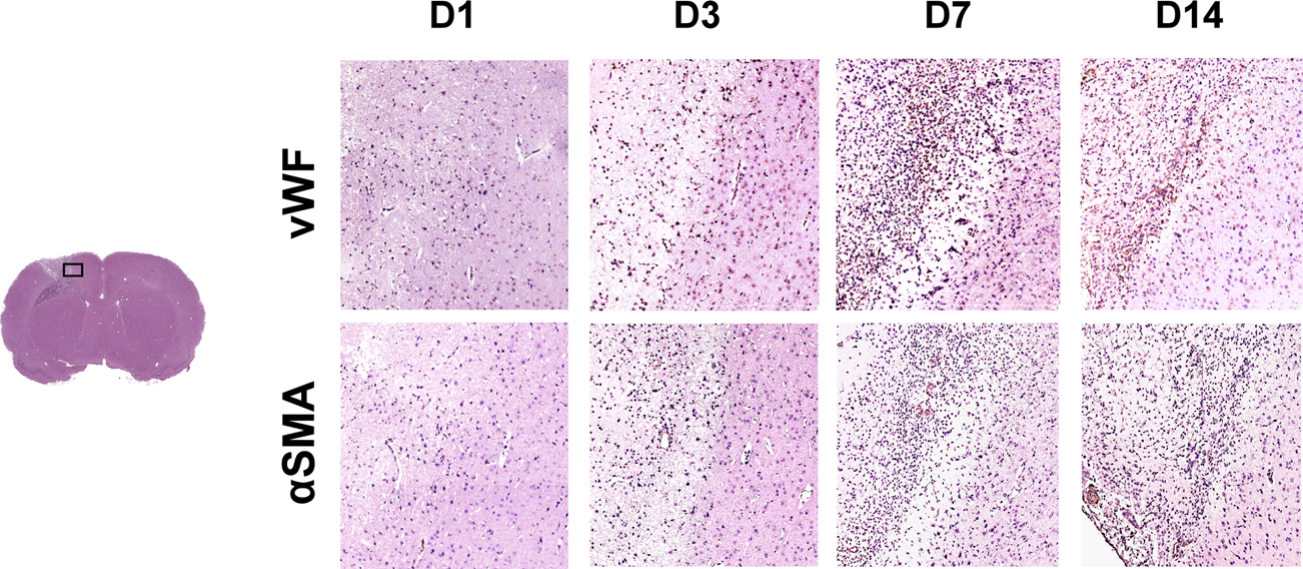
Immunohistochemical staining of vWF and αSMA at day 1, 3, 7, and 14 after stroke induction. Scattered vWF–vessels were observed in the margin of infarct zone at day 3, and the number of vWF- and αSMA-positive vasculature began to increase at day 7. At day 14, neovascularization notably occurred in the core of infarct zone

As shown in Fig. 4, there were scattered vWF (a marker of endothelial cell) positive vessels in infarct boundary at day 3, and the number of capillaries and αSMA (a marker of vascular smooth muscle cells) positive vessels significantly increased along the infarct margin at day 7. Further, neovascularization was detected in the core of infarct lesion at day 14. These results suggested that angiogenesis and vasculogenesis occurred in the regions of infarct and peri-infarct lesion within 2 weeks after photochemically induced stroke.

### The Dynamic Evaluation of Brain Edema and CBF

**Fig. 5:**
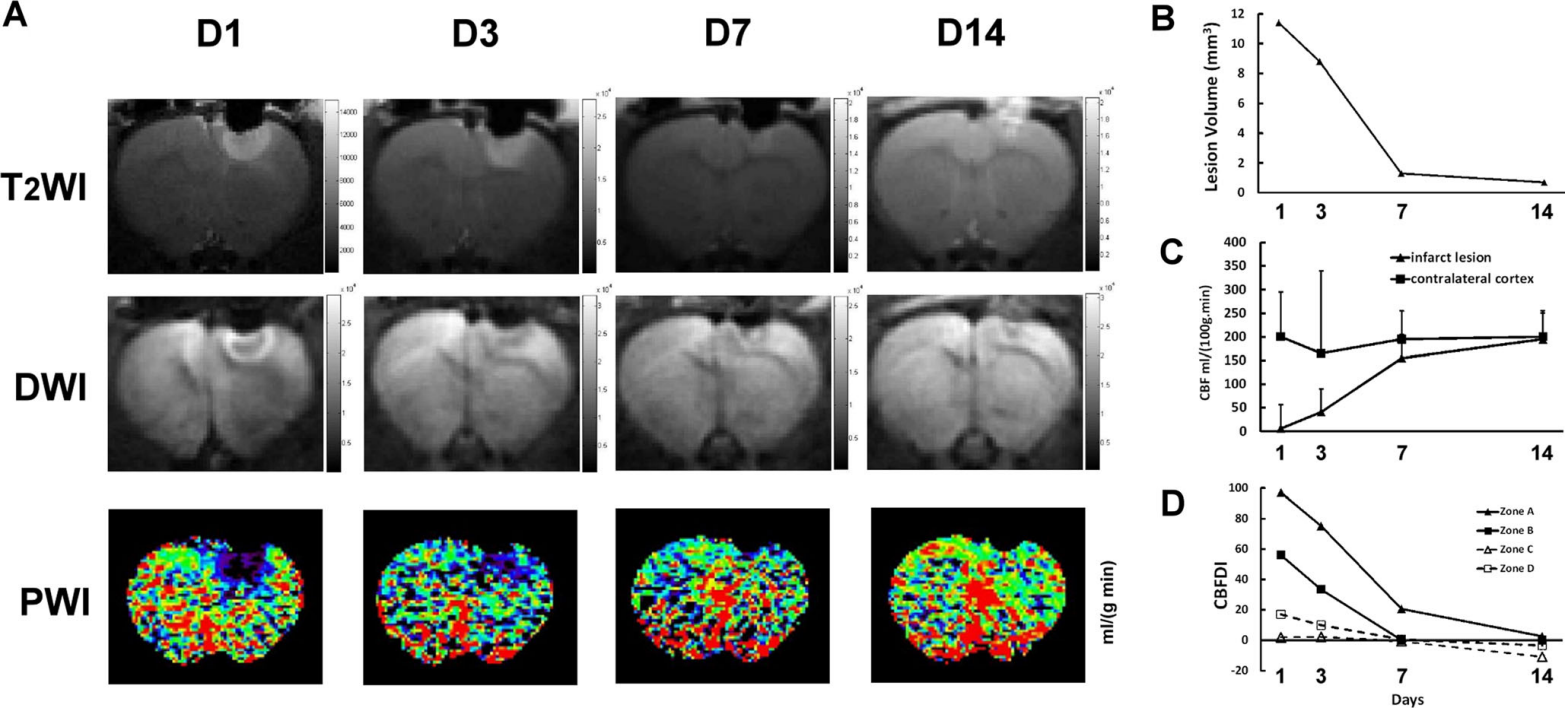
Sequential images of T_2_WI, DWI, and PWI in stroke lesion on the right somatosensory cortex and CBF analysis. **a** Images of T_2_WI, DWI, and PWI at day 1, 3, 7, and 14 after ischemia induction demonstrated the evolution of vasogenic edema and cytotoxic edema with time. **b** Evolutional changes and CBFDI, which was calculated from the regional CBF obtained from the PWI of the infarct lesion (zone A), peri-lesional region (zone B, one-pixel width surrounding the infarct lesion), ipsilateral remote cortex (zone C), and hippocampus (zone D). It suggests that CBF of zone B improved more significantly than that of zone A from day 7 after induction

To monitor the evolutional brain edema and hemodynamic change of photothrombotic stroke, T_2_WI, DWI, and PWI were performed on rats at day 1, 3, 7, and 14 after stroke induction. The ischemic lesion showed a hyperintensity in T_2_WI with sharp margin at day 1, and the signals gradually declined afterward (Fig. 5a). Quantitative analysis indicated that the lesion volume declined from 11.4 mm^3^ at day 1 to 0.7 mm^3^ at day 14 (Fig. 5b). In DWI, restriction of water diffusion was observed at the margin of stroke shown as an intense halo at day 1 and day 3, but not in the core of the lesion which showed a hypointense signal instead. At day 7, the halo was getting smaller. The hypointense lesion almost completely disappeared at day 14 (Fig. 5a). As T_2_WI and DWI have been the reliable methods for investigation of vasogenic edema and cytotoxic edema, respectively [20, 21, 23], gradual reduction of hypointense signal of T_2_WI and DWI suggested the improvement of brain edema, which contributed to decrease of infarct volume. PWI at day 1 after photochemical induction demonstrated a region of compromised CBF on arterial spin labeling (ASL) corresponding to the photothrombotic infarct lesion (6 ± 51 ml/100 g/min vs 200 ± 95 ml/100 g/min of contralateral cortex), and slightly decreased CBF in the remote cortex of ipsilateral hemisphere. The CBF compromised zone was getting smaller with time. At day 14, a tiny infarct core was still noted, suggesting a persisted damage of brain tissue. CBF of the ischemic region recovered to 195 ± 55 ml/100 g/min (Fig. 5c). CBFDI of the infarct region (zone A) and the peri-infarct region (zone B) declined rapidly at day 7. CBFDIs of the infarct region and the peri-infarct region showed no significant difference with the contralateral cortex at day 7 and day 14, respectively. CBFDI of ipsilateral remote cortex (zone C) and hippocampus (zone D) closed to zero means CBF almost identical to that of the contralateral hemisphere throughout the whole study of 14 days (Fig. 5d). In summary, the MRI results revealed progressive shrinkage of stroke volume at the periphery of the infarct lesion early after stroke and almost complete recovery of CBF within 2 weeks after stroke induction. Moreover, the remarkable improvement of CBF in infarct lesion and peri-infarct region at day 7 might be related to the synchronous enhancement of angiogenesis and vasculogenesis.

### Glucose Metabolism of Stroke Lesion by ^18^F-FDG PET Imaging

**Fig. 6:**
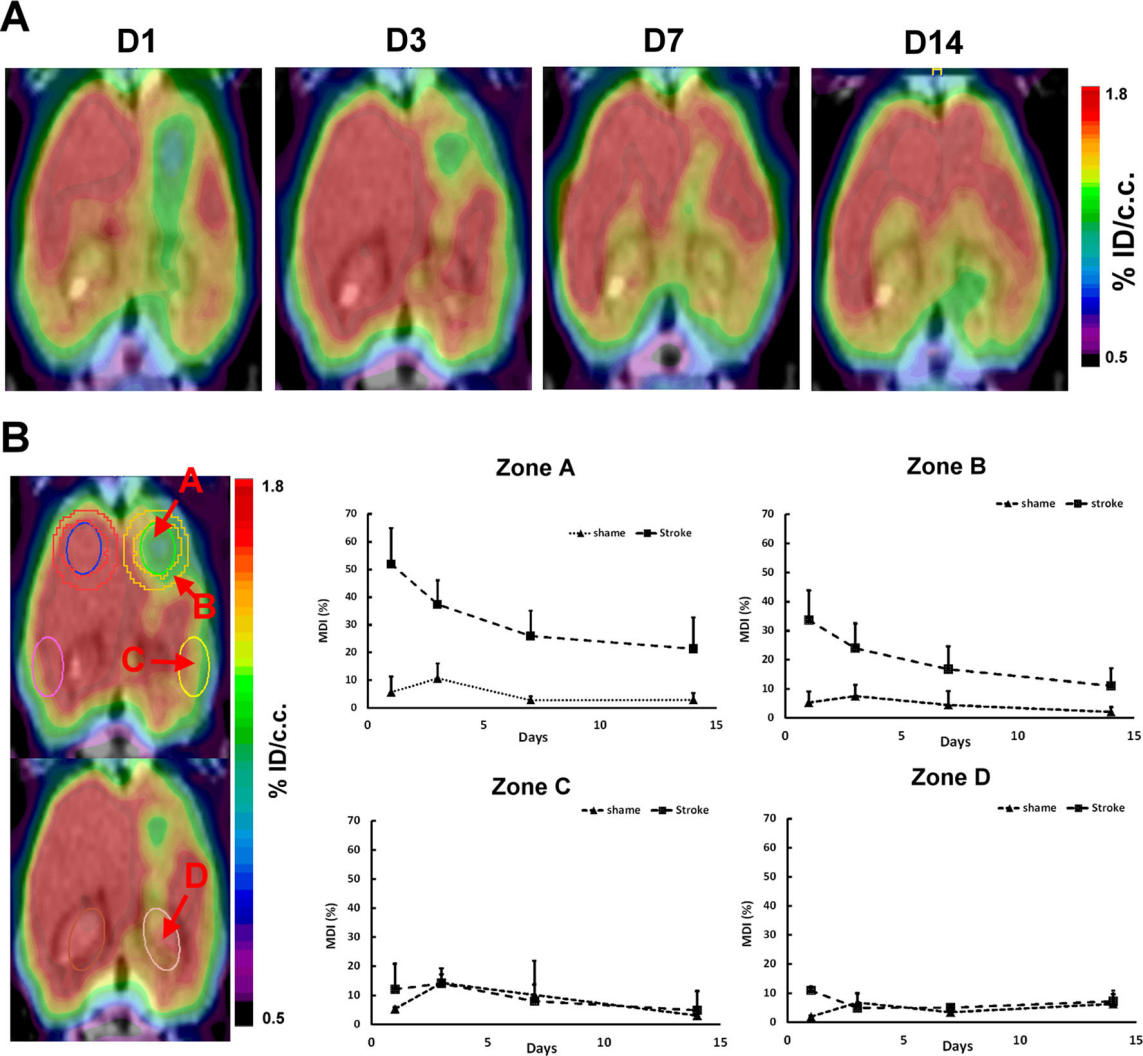
Sequential ^18^F-FDG PET imaging of rats with photochemical thrombosis on the right somatosensory cortex. **a** Transaxial tomographic images at day 1, 3, 7, and 14 after stroke induction. **b** Metabolic difference index (MDI) at ROIs of infarct zone (zone A), one-pixel width surrounding the lesion (zone B, one-pixel width surrounding the infarct lesion), remote region of the ipsilateral cortex (zone C), and hippocampus (zone D) showed at each time point. There was significant decrease in MDIs with time in zone A and zone B in 14 days (*p* < 0.05). Stroke rats, *n* = 5; shame rats, *n* = 3

To investigate the metabolic change of the photochemically induced brain stroke with time, ^18^F–FDG PET imaging was performed at day 1, 3, 7, and 14 after induction of stroke. As shown in Fig. 6a, a remarkably reduced ^18^F-FDG uptake was noted at the site of infarct lesion and moderately reduced uptake in the remote region of ipsilateral hemisphere at day 1 following induction of stroke. At day 3, the cortical metabolic defect partially recovered in the outer region of the ischemic zone and almost completely recovered in the remote cortex. At day 14, the ^18^F-FDG uptake at the infarct site almost completely recovered. MDI was calculated to monitor the change of glucose utilization in the infarct lesion (zone A), in the region at one-pixel width surrounding the lesion (zone B), in the remote region of the ipsilateral cortex (zone C), as well as in the hippocampus (zone D). MDIs of zones A and B were decreasing with time, indicating that the glucose consumption was gradually restored at both the infarct region and the margin (Fig. 6b). There was significant decrease in MDIs with time in zone A and zone B in 14 days (ANOVA, *p* < 0.05). From day 7 to day 14, MDI of zone A decreased by 10 % compared to 20 % decrease in zone B, indicating that the recovery of glucose metabolism at the margin of the infarct is better than that in the infarct core. There was no significant statistical difference of MDI observed for 14 days in zones C and D (ANOVA, *p* > 0.05), suggesting no obvious compromise of glucose metabolism observed in the ipsilateral remote cortex and hippocampus. Using photothrombotic method, infarct induction at somatosensory cortex did not interfere the glucose utilization in the remote ipsilateral cortex and hippocampus and contralateral cortex as well.

### Evolution of BBB Permeability in Photothromboic Stroke

**Fig. 7:**
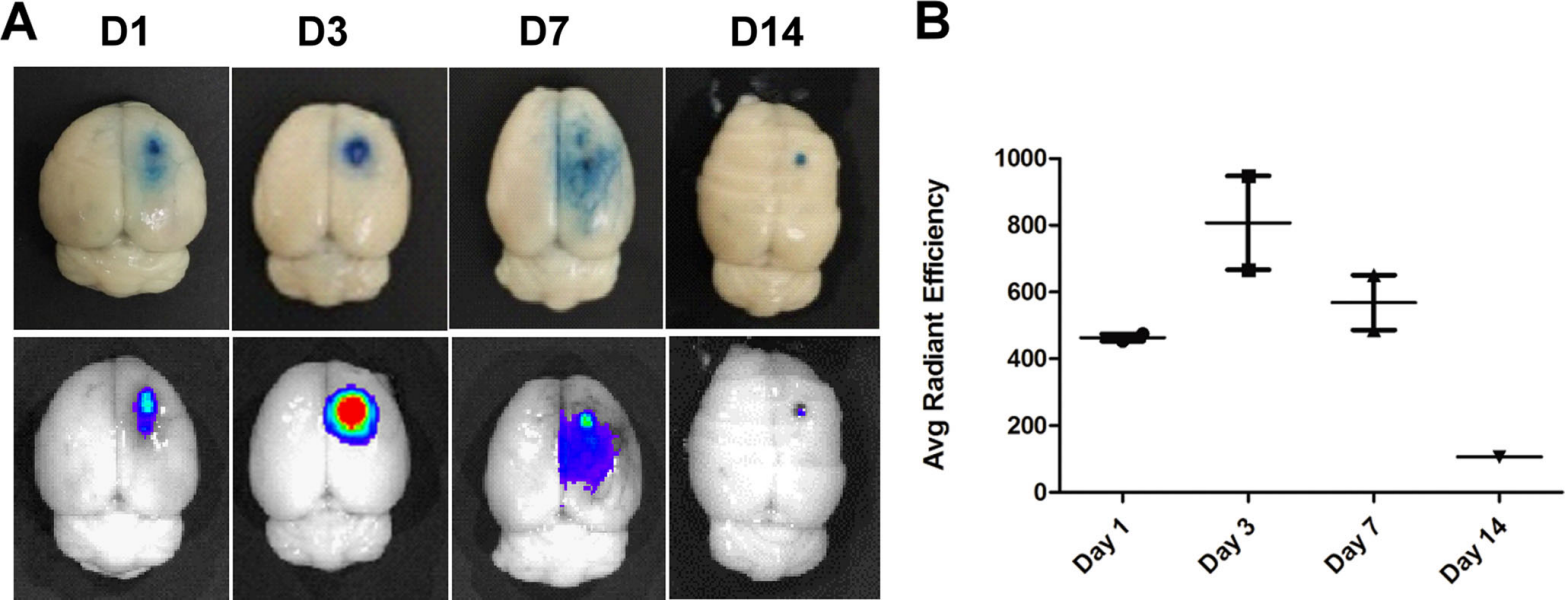
Detection of blood-brain barrier disruption by EB staining. **a** Representative images of EB staining and fluorescence imaging of rat brain at day 1, 3, 7, and 14 after ischemia induction. **b** Quantitative analysis of EB fluorescence at the ROIs of ischemic region (*n* = 3 at each time point). It suggested that the leakage of BBB was most remarkable at day 3 and was subsequently decreasing with time

Evidence indicates that BBB disruption occurs after ischemic stroke and leads to the leakage of normally excluded substances into interstitial fluid of the brain. To evaluate the extent of BBB disruption and its restoration, we performed the EB staining and ex vivo fluorescence imaging at day 1, 3, 7, and 14 post-stroke. Figure 7a demonstrated an infarction core surrounded by an EB-stained zone in the somatosensory cortex at day 1 after photochemical induction. Subsequently, the leakage of BBB gradually increased at day 3 and began to restore at day 7; a small EB-stained lesion still remained at day 14. The ex vivo fluorescence imaging showed a high intensity fluorescence corresponding to the EB-stained ischemic lesion. The fluorescence signals were much less intense and less extensive at day 14. Quantitative analysis of EB fluorescence at the ROIs of ischemic region showed a markedly reduced signal intensity at day 14 compared to the highest intensity at day 3 (Fig. 7b, *p* < 0.05). Fluorescent signal at peri-infarct region was much lower than that at infarct core throughout 7 days and greatly reduced to a background level while the infarct core still showed a high signal at day 14. These results suggest a favorable self-repair of BBB in 14 days after photothrombotic stroke.

## Discussion

**Fig. 8:**
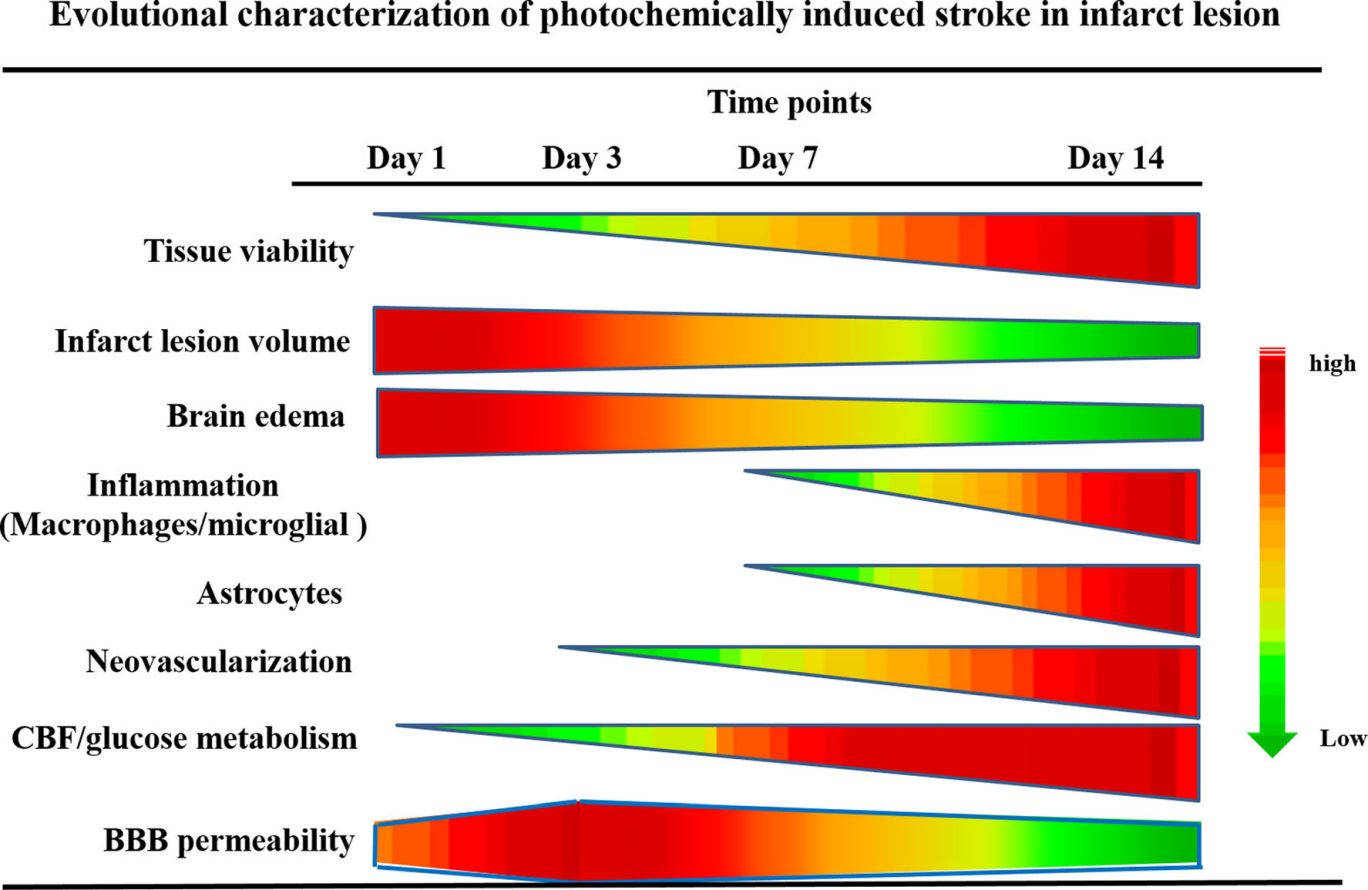
Evolutional characterization of photochemically induced stroke. The changes of tissue viability, infarct lesion volume, brain edema, inflammatory cells and astrocyte infiltration, neovascularization, CBF, glucose metabolism, and BBB permeability at each time point were summarized

In the present study, we have successfully assessed the evolutional changes of cellular viability in the lesion, infarct volume, brain edema, infiltration of inflammatory cells and astrocytes, neovascularization, CBF, glucose metabolism, and BBB permeability (Fig. 8). To the best of our knowledge, this is the first study conducted using ^18^F-FDG/PET and MRI to longitudinally evaluate the metabolic and hemodynamic changes of the brain in the rat model of photochemically induced cerebral infarction.

The method applied in current study was categorized as “end-artery occlusion in the cortex” described by Chen et al. [23]. It is the classical and simplest method of photochemically induced stroke model. Although this method can be achieved with intact skull, however, to avoid the scatter and reflection of light when illuminating through skull, a cranial window was made before illumination. In this way, the infarction is anticipated to be more sharply demarcated and more consistent in lesion size than using intact skull. Also, the volume of the ischemic lesion can be controlled by manipulating the intensity of irradiating light and the size of irradiated zone in the cranial window [11]. In the current study, a laser beam of 532 nm wavelength was applied instead of arc beam irradiated system with 560 nm wavelength. Less intensity of the irradiating light causing less severity of the induced stroke may account for the short period of ipsilateral hypometabolism and lack of compromised glucose utilization in contralateral cortex.

To confirm the success of photochemical induction, we applied LSCI through the cranial window of the rat before and after thrombosis was induced to assess the occlusion of cerebral vasculature caused by photochemically induced thrombosis. Laser speckle imaging is useful to assess cerebral vasculature and CBF noninvasively with high temporal and spatial resolution [28]. It has been used to study changes of CBF after distal middle cerebral artery ligation in mice [31]. Upon photochemical induction, LSCI demonstrated blockade of the majority of blood vessels in the illuminated region, indicating successful induction of cerebral ischemia and/or infarction.

In the evaluation of tissue viability and morphologic change in infarct lesion, a wedge-shaped TTC-negative area appeared at day 1 after stroke induction and the size gradually reduced through day 3 to day 7 and was eventually not detectable at day 14. The result of H&E stain showed increased number of infiltrated cells in infarct region since day 3, and some cells were detected in the infarct core at day 14. The identity of these infiltrated cells was further confirmed to be macrophages, glial, endothelial, and smooth muscle cells, but not neuronal cells by IHC staining. Infiltration of cells into the infarct region caused the gradual shrinkage of TTC-negative area.

Inflammation is strongly linked to the development of stroke. Macrophages and microglia, two important types of inflammatory cells in response to stroke, have significant effect on removing debris and activating inflammatory cascades involved in repairing tissue damage [32, 33]. In the present study, IHC staining showed that CD68 positive macrophages/microglia and GFAP-expressing astrocytes notably accumulated along the boundary of infarct lesion at day 14, forming a belt consisting of inflammatory cells and astrocytes. Simultaneously, some microglia/macrophages and astrocytes were detected in the core of infarct lesion. Previous reports suggested that astrocytes produce pro-inflammatory cytokines and chemokines which subsequently recruit microglia/macrophages for dead cell clearance [34, 35]. However, excessive inflammation may have adverse effect, i.e., inducing free radicals which are harmful to neuronal cells [36]. Also, excessive thickness of astrocyte-associated scar blocks neuronal cells from migrating into the injured site. This evidence further supports our result in which neuronal cells were not detectable in the ischemic lesion throughout 14 days after stroke induction.

Vasogenic edema examined by T_2_WI is strongly correlated with BBB disruption after stroke [37, 38]. This was also observed in present study. Hyperintensity in T_2_WI at day 1 and day 3 post-stroke was paralleled with the intensity of EB fluorescence, indicating the linkage between vasogenic edema and BBB permeability in photothrombotic stroke. Serial DWI in the current study revealed an intense halo located at the periphery of the stroke lesion seen on T_2_WI. This hyperintense halo seen on DWI indicated the zone with cytotoxic edema and began to gradually reduce since day 3 post-stroke. MR images of photochemical stroke in a previous report also showed early increase in T_2_ signal and decreased diffusion of water, indicating the simultaneous development of substantial vasogenic edema and ischemic infarction [39]. This pattern is different from that seen in human stroke, where infarcts develop with cytotoxic edema, followed by a vasogenic edema which is delayed by several hours [3].

The PWI demonstrated a region of absent CBF at the infarct site as well as the edematous region. CBFDI revealed progressive restoration of CBF in the infarct zone and returned to normal at day 14. CBF in the peri-infarct zone was less compromised than that in the infarct zone and returned to normal at day 7. The status of CBF was further confirmed and well correlated with IHC results which showed dilated vessels close to the infarct core at day 3 and neovascularization at day 7 to 14.

In current study, progressive recovery of glucose utilization was noted at the infarct lesion, peri-infarct zone, and ipsilateral remote cortex. CBF recovery and progressive cell infiltration subsequently causing enhancement of glucose utilization may partially account for the rapid recovery of ^18^F-FDG uptake in the infarct zone and its vicinity. Analysis of the MDI is more sensitive than visual inspection of hypometabolic lesion. MDI at day 14 revealed relatively higher restoration of glucose consumption at the peri-infarct zone than that in the infarct core (10 vs 20 %, respectively). Several preclinical studies of small animal ischemic stroke models have consistently revealed an increased ^18^F-FDG uptake in the peri-infarct regions due to the effects of activation of glucose transporters, hexokinase, and neuroinflammation [40]. Previous study which used the model of photothrombotic MCAo in rats showed that ^18^F-FDG uptake in the peri-ischemic areas was comparable to the normal brain regions at day 1 and 3 and notably elevated at days 7 and 14 [41]. In the present study, glucose utilization in the peri-infarct region was decreased compared to that in the normal region throughout 14 days after stroke induction. However, progressive recovery of glucose utilization in this region was noted. These different results of glucose metabolism in peri-infarct area might attribute to different animal stroke models, time of evaluation, and imaging techniques.

It is commonly agreed that no salvageable penumbral tissue exists in the model of photochemically induced stroke. This disadvantageous phenomenon of this model is different to that of other embolic models where penumbra is much more like those in human stroke and much larger. Hilger and colleagues named peri-infarct zone of photothrombotic stroke as “region-at-risk,” where a little viable cells, low energy metabolism, and vasogenic edema existed [42]. In present study, the number of inflammatory cells and astrocytes, as well as neovascularization, gradually increased in peri-infarct region. At day 14, a belt consisting of inflammatory cells and astrocytes was observed surrounding the infarct lesion. In addition, vasogenic edema and cytotoxic edema progressively reduced, and CBF and glucose metabolism gradually improved. The leakage of EB dye at the peri-infarct zone significantly reduced than that in the infarct core. These data suggests the recoverable BBB leakage, neovascularization, CBF, and glucose metabolism at peri-infarct region rather than at infarct core.

Although we have longitudinally characterized the pathophysiological changes of photothrombotic brain ischemia by multiple imaging modalities and cellular immunostaining, some limitations should be addressed in the current study. First, photochemically induced occlusion occurred in vessels within the irradiation area, where the mechanism is different from that of clinical ischemic stroke which was usually caused by an embolus or two. Only a little or no local collateral flow/reperfusion and ischemic penumbra occur in this model. Despite that the cellular changes in this model are still evolving and follow a similar trend to those of other occlusive models, the pathomechanism is different from those seen in human stroke. Second, evaluation was not performed at earlier time points (3–12 h post-stroke) due to poor animal condition early after stroke induction for ^18^F-FDG/PET and MRI examination. Sequential assessment of the evolutional change of photochemically induced rat stroke model during the first 24 h following stroke will be carried out in the future study. Third, we did not perform quantitation of blood flow of regional cerebral vasculature by LSCI because the measurement of CBF is limited to the superficial cortex. Fourth, the experiments of multi-modality imaging were performed on the same animal as possible as we could for serial imaging protocol. However, to avoid being anesthetized for a long period of time on the same animal, PET and MRI imaging were not performed on the same day. Despite these drawbacks and limitations of this model, the advantages of easy manipulation, highly reproducible, lesion controllable, and low mortality have made this method comprehensively applied. Further, the consistent lesion made in the cortex where cellular changes evolve and follow a similar trend to those of the occlusive models makes it a suitable model for the study of neurorestorative therapy of pharmaceuticals or stem cells. In the scope of translational purpose, this method had been applied on animals other than rodents, such as rabbits and pigs [43–46].

## Conclusion

The evolution of photochemically induced stroke model in rats has been longitudinally characterized for 14 days by ^18^F-FDG/PET, MRI, IVIS, histopathology, and immunohistochemistry examination. Within 14 days after stroke induction, we found the occurrence of early brain edema, infiltration of inflammatory cells and astrocytes, neovascularization in the infarct core and peri-infarct zone, improvement of CBF, glucose metabolism, and BBB permeability. These serial changes characterized in this study provide better understanding of cerebral ischemia and are highly beneficial to the development of therapeutic strategies for ischemic stroke.

## Abbreviation

BBB, blood-brain barrier; BSA, bovine serum albumin;^14^C-2-DG, ^14^C-2-Deoxy-D-Glucose;CBF, cerebral blood flow; CBFDI, difference indexes;CPMG, Carr, Purcell, Meiboom, Gill; CT, computed tomography; DW, diffusion weighted; DWI, diffusion-weighted image (imaging); EB, Evans blue;^18^F-FDG, ^18^F-2-deoxy-glucose; FOV, field of view; GFAP, glial fibrillary acid protein; H&E, hematoxylin-eosin; IHC, immunohistochemistry; IVIS, In Vivo Imaging System; LSCI, Laser speckle contrast imaging;MCAo, middle cerebral artery occlusion; MDI, metabolic difference index; MRI, magnetic resonance imaging; OSEM, ordered subsets expectation maximization; PBS, phosphate buffer saline; PET, positron emission tomography; PWI, perfusion-weighted image (imaging); RF, radio frequency; ROI, Region of Interest; S.D., standard deviation; αSMA, alpha smooth muscle actin; TBS, tris-HCL buffered solution; TIR, inversion recovery time; TTC, 2, 3, 5-triphenyl tetrazolium chloride; TE, echo time; TR, repetition time; T2W, T2 weighted; T2WI, T2-weighted image (imaging); VOIs, volume of interests; vWF, von Willebrand Factor;%ID/cm^3^, percent injected dose per c.c;
